# Blood Alcohol Level as a Predictor of Withdrawal Severity

**DOI:** 10.7759/cureus.44463

**Published:** 2023-08-31

**Authors:** Megan Mayer, Amgad Masoud

**Affiliations:** 1 Department of Internal Medicine, University of Missouri-Kansas City School of Medicine, Kansas City, USA

**Keywords:** alcohol withdrawal treatment, alcohol withdrawal seizures, withdrawal at high blood alcohol level, severe alcohol withdrawal, blood alcohol concentration, prognosis of alcohol withdrawal, blood alcohol level, alcohol withdrawal syndrome

## Abstract

Few studies have explored the correlation between the severity of alcohol withdrawal and blood alcohol level at the time of admission. Specifying prognostic factors for life-threatening withdrawal necessitating inpatient pharmacologic management over the course of days would be useful to identify at-risk patients at the time of admission. Hence, we present the case of a 34-year-old Caucasian male with a past medical history of poly-substance abuse who has presented to our emergency department 11 times over the past four years with a mean blood alcohol level (BAL) of 287 mg/dL upon withdrawal. BAL at the time of withdrawal is highly variable depending on the chronicity of abuse; however, a BAL this elevated is highly unusual and indicative of severe and long-term use. While in the unit at this admission, the patient's BAL was 437 and his withdrawal symptoms were severe, necessitating ICU admission and strong sedating medications to control his symptoms. Even after these interventions, he still demonstrated severe withdrawal symptoms including full body tremors, vital sign instability, and continuous visual, auditory, and tactile hallucinations. This patient presents an interesting case of severe alcohol withdrawal at an abnormally elevated blood alcohol level progressing to a prolonged withdrawal course in the ICU. Alcohol level at the time of withdrawal could be a helpful predictor of the course of severity of alcohol withdrawal; however, more studies are required to prove this relationship.

## Introduction

Alcohol withdrawal syndrome (AWS) is a condition affecting individuals with alcohol use disorder, occurring after an abrupt reduction or discontinuation from their normal level of daily alcohol intake. Signs of alcohol withdrawal have been well studied and range from mild disease, presenting with diaphoresis, agitation, and anxiety, to life-threatening disease characterized by delirium tremens (DT), catatonia, respiratory depression, metabolic derangements, and withdrawal seizures [1]. The gold standard treatment of AWS is short-acting benzodiazepines; however, more involved measures are indicated for severe cases. Various tools are accepted to predict risk for one of the most feared complications of withdrawal, DT, such as the Prediction of Alcohol Withdrawal Severity Scale [2]. Notable factors that are strongly associated with the development of complicated severe withdrawal are previous episodes of DT and the course of previous withdrawal [3].

Few studies have explored the relationship between the blood alcohol concentration (BAC) on admission and the severity of the withdrawal course. Some that have studied this association have found promising data that high BAC on admission is correlated with withdrawal severity. Specifically, a retrospective cohort study that included 185 patients across two institutions found that alcohol levels at admission correlated with withdrawal severity over the first 48 hours of admission. This finding remained statistically significant even after adjustment for other variables [4]. Still, most prognostic tools do not incorporate BAC as a factor. Instead, they rely on other signs such as past episodes of withdrawal to assess the risk for severe withdrawal and subsequent DT. This requires subjective information from the patient to complete the risk assessment, which may be difficult if the patient is obtunded on admission. We present the case of a chronic alcohol user with recurrent severe withdrawal at an abnormally high blood alcohol level, necessitating recurrent prolonged ICU admissions.

## Case presentation

We present a case of a 34-year-old Caucasian male with a past medical history of poly-substance abuse, unspecified seizure disorder noncompliant to Keppra, multiple prior past hospitalizations for alcohol withdrawal, and a subjective history of withdrawal seizures, who presented to the emergency department at the time of acute alcohol withdrawal with a blood alcohol level of 437 mg/dL at the onset of withdrawal (Table 1). His baseline alcohol intake was reported at about one gallon of vodka per day, and the patient reported his last drink was 12 hours prior to presentation. This patient also disclosed a history of smoking methamphetamine, most recently used one week before presentation.

**Table 1:** Laboratory Values at Admission AST: aspartate transaminase; ALT: alanine transaminase; C: critical; H: high; L: low; Na: Sodium; K: Potassium; BUN: blood urea nitrogen; MCV: mean corpuscular volume; HDL: high-density lipoprotein; LDL: low-density lipoprotein; VLDL: very low density lipoprotein

Chemistry	Hematology
Na 140	WBC 9.70
K 4.4	RBC 4.10: L
Cl 106: H	Hemoglobin 13.0: L
CO2 26	Hematocrit 38.3: L
Anion Gap 8: L	MCV 93.5
Glucose 77	Platelet 390
BUN 15	Toxicology
Creatinine 0.71: L	Alcohol, serum 437: C
BUN/Creatinine Ratio 21.1	Alcohol, serum (previous admission) 309: C
Calcium 8.5: L	Alcohol, serum (previous admission) 214: H
Bilirubin Total 0.3	
Alkaline Phosphatase 63	
AST 44: H	
ALT129: H	
Cholesterol 115	
Triglyceride 52	
HDL 59	
LDL 46: L	
VLDL 10	

On presentation, the patient reported symptoms of early withdrawal including tremors, palpitations, headache, diaphoresis, abdominal pain, as well as visual and auditory hallucinations. Initial vital signs showed tachycardia up to 111 beats per minute and blood pressure of 143/103 mmHg. On physical examination, the patient appeared in moderate distress with constant tremors. The patient was alert and oriented. Laboratory values showed aspartate aminotransferase (AST) of 44 U/L and alanine transaminase (ALT) of 129 U/L (Table 1). Urine drug screen was positive for cannabinoids. Pharmacologic intervention in the emergency department consisted of restarting home Keppra 500 mg twice daily, initiation of Clinical Institute Withdrawal Assessment for Alcohol (CIWA) protocol, chlordiazepoxide 50 mg/day, thiamine 100 mg/day, and folic acid 1 mg/day. Shortly thereafter, his condition began to deteriorate and he was admitted to the ICU for further management.

The patient remained in the ICU for the remainder of his stay, as he was at high risk for DT and was critically ill necessitating a continuous dexmedetomidine infusion. The patient’s CIWA score was persistently greater than 20 throughout his admission requiring large doses of benzodiazepines, only decreasing on day five of admission (Table 2). On the sixth day, the patient left against medical advice and had the capacity to do so, although he still reported some visual hallucinations and tremors. These symptoms were much improved from the day of admission and were not recorded in CIWA on the day of discharge. At each of this patient’s previous visits due to withdrawal, he presented with a CIWA score greater than 20, requiring admission to the ICU for multiple days. Each visit required large numbers of benzodiazepines over the course of multiple days. 

**Table 2:** Clinical Institute Withdrawal Assessment for Alcohol-Revised (CIWA-Ar) Scores

AM values	Day 1	Day 2	Day 3	Day 4	Day 5	Day 6
	Nausea/vomiting 4	Nausea/vomiting 3	Nausea/vomiting 3	Nausea/vomiting 2	Nausea/vomiting 0	Nausea/vomiting 1
	Tremors 5	Tremors 7	Tremors 5	Tremors 4	Tremors 1	Tremors 6
	Anxiety 4	Anxiety 3	Anxiety 4	Anxiety 4	Anxiety 1	Anxiety 2
	Agitation 4	Agitation 1	Agitation 3	Agitation 4	Agitation 1	Agitation 2
	Paroxysmal sweats 0	Paroxysmal sweats 3	Paroxysmal sweats 2	Paroxysmal sweats 1	Paroxysmal sweats 0	Paroxysmal sweats 3
	Orientation 0	Orientation 3	Orientation 0	Orientation 0	Orientation 0	Orientation 0
	Tactile Disturbances 3	Tactile Disturbances 3	Tactile Disturbances 1	Tactile Disturbances 0	Tactile Disturbances 0	Tactile Disturbances 0
	Auditory Disturbances 0	Auditory Disturbances 3	Auditory Disturbances 1	Auditory Disturbances 2	Auditory Disturbances 0	Auditory Disturbances 0
	Visual Distrubances 0	Visual Distrubances 3	Visual Distrubances 1	Visual Distrubances 2	Visual Distrubances 0	Visual Distrubances 0
	Headache 0	Headache 3	Headache 3	Headache 1	Headache 0	Headache 0
	CIWA-Ar Total Score: 20	CIWA-Ar Total Score: 32	CIWA-Ar Total Score: 23	CIWA-Ar Total Score: 20	CIWA-Ar Total Score: 3	CIWA-Ar Total Score: 14
PM values	Day 1	Day 2	Day 3	Day 4	Day 5	Day 6
	Nausea/vomiting 4	Nausea/vomiting 2	Nausea/vomiting 4	Nausea/vomiting 0	Nausea/vomiting 0	Nausea/vomiting 0
	Tremors 4	Tremors 2	Tremors 4	Tremors 7	Tremors 1	Tremors 5
	Anxiety 5	Anxiety 2	Anxiety 3	Anxiety 4	Anxiety 3	Anxiety 4
	Agitation 5	Agitation 1	Agitation 3	Agitation 4	Agitation 0	Agitation 5
	Paroxysmal sweats 6	Paroxysmal sweats 1	Paroxysmal sweats 3	Paroxysmal sweats 0	Paroxysmal sweats 0	Paroxysmal sweats 2
	Orientation 3	Orientation 3	Orientation 3	Orientation 0	Orientation 0	Orientation 0
	Tactile Disturbances 2	Tactile Disturbances 2	Tactile Disturbances 2	Tactile Disturbances 0	Tactile Disturbances 0	Tactile Disturbances 0
	Auditory Disturbances 0	Auditory Disturbances 2	Auditory Disturbances 2	Auditory Disturbances 1	Auditory Disturbances 0	Auditory Disturbances 0
	Visual Distrubances 2	Visual Distrubances 1	Visual Distrubances 2	Visual Distrubances 1	Visual Distrubances 0	Visual Distrubances 0
	Headache 3	Headache 2	Headache 4	Headache 1	Headache 0	Headache 0
	CIWA-Ar Total Score: 35	CIWA-Ar Total Score: 18	CIWA-Ar Total Score: 30	CIWA-Ar Total Score: 18	CIWA-Ar Total Score: 4	CIWA-Ar Total Score: 16

## Discussion

AWS is a potentially life-threatening diagnosis; therefore, patients presenting to an inpatient service with a high blood alcohol concentration must be followed closely to prevent severe complications. Aggressive pharmacological management may not be sufficient to prevent all withdrawal symptoms, especially if the course of withdrawal is severe, as seen in this patient.

Blood alcohol level at the time of presentation or at the time of withdrawal may be a useful clinical prognostic factor to predict the severity of withdrawal, and reason to initiate more intensive pharmacological therapy. In a retrospective cohort study that assessed 185 patients admitted for alcohol withdrawal, alcohol level at the time of admission was significantly associated with withdrawal severity [4]. Severity was measured by the amount of chlordiazepoxide administered within the first 48 hours of admission, institutional withdrawal severity assessment score, and severity of tremor. This study recommended creating a threshold of 150 mg/dL on admission to help identify patients most at risk for severe adverse outcomes. In this study, patients’ BACs were measured regardless of whether the patient presented with acute intoxication or was already in withdrawal. 

The current report demonstrates the importance of close attention to the time that BAC is measured. The BAC of the patient in this report was 437 mg/dL 12 hours after his last drink, while he was already in withdrawal. This measurement may provide different clinical utility than a BAC measured immediately after the first sign of alcohol withdrawal. In order to identify the most accurate threshold for blood alcohol level to be used clinically, more data is needed on patients’ BACs at the onset of withdrawal, as well as at the time of presentation to the hospital. Although a few studies, as mentioned, have studied this relationship, blood alcohol level either on admission or at the start of withdrawal is not yet being utilized as a prognostic factor for the course of withdrawal and development of severe complications. More data collection is indicated to determine its clinical utility as part of a larger prediction assessment tool. 

Most withdrawal occurs at a low or normal blood alcohol level. Some cases have been studied where patients begin to withdraw at abnormally high blood alcohol levels, up to 150 mg/dL [5]. Our patient is unique in the sense that in each repeat withdrawal admission, he presents with a blood alcohol level well above the range of previously reported cases. In fact, withdrawal commonly occurs at a blood alcohol concentration of zero, as demonstrated in an observational study of 539 cases of alcohol withdrawal, where 48 of 539 patients presented with a blood alcohol level of zero. In that study, the median BAC on admission was 160 mg/dL and withdrawal began at a median of five hours after admission [6]. This delayed withdrawal indicates that blood alcohol level typically decreases even further before withdrawal precipitates, which was not the case for our patient whose syndrome began at a level of 437 mg/dL.

Withdrawal at high BAL is uncommon; however, another explanation for withdrawal at high levels has been studied. Benzodiazepines and alcohol act on the same receptor and are cross-reactive, meaning that the effects of these substances are additive. Benzodiazepine withdrawal may begin and mirror a withdrawal syndrome, even as the patient remains at a high or baseline blood alcohol level [5]. This syndrome is to be considered when a patient presents with a withdrawal syndrome and high blood alcohol level. 

Because of chronic alcohol use by the patient in the current report, it is likely that his blood alcohol level is chronically elevated. Although a blood alcohol level of 437 mg/dL is still abnormally high, in this particular patient, it was the level at which he went into withdrawal. This level was measured 12 hours after his last drink. The patient presented multiple times earlier in severe withdrawal with a blood alcohol level that was consistently abnormally elevated. This is a highly unusual presentation, which may be correlated to the consistent severity of his withdrawal. Even after large numbers of benzodiazepines and sedating pharmacotherapy, this patient still suffered a severe and prolonged withdrawal, on par with his previous admissions. This unique presentation encourages more studies to determine if blood alcohol level can be utilized as a prognostic factor in anticipation of the management of severe acute alcohol withdrawal. 

## Conclusions

We described a case of recurrent severe alcohol withdrawal that was always associated with a very high blood alcohol level at presentation. The patient almost always progressed to a severe withdrawal course lasting more than a week requiring intensive pharmacological therapy in the ICU. The patient’s alcohol level at the time of withdrawal may help to predict the course of severity of alcohol withdrawal. 
